# The Cognitive Toll of Digital Overuse on Migraine in Young Adults

**DOI:** 10.7759/cureus.99236

**Published:** 2025-12-14

**Authors:** Ivan Andrei, Eliza Cristina Ghita, Gabriel-Vladimir C Ilie, Marius P Iordache, Irina M Protosevici

**Affiliations:** 1 Neurology, Ilfov County Clinical Emergency Hospital, Bucharest, ROU; 2 Neurology, Spitalul Clinic Judetean de Urgenta Ilfov, Bucharest, ROU; 3 Faculty of Medicine, Titu Maiorescu University, Bucharest, ROU; 4 Neurology, Titu Maiorescu University, Bucharest, ROU

**Keywords:** digital usage, migraine disorder, migraine in young adults, migraines and headaches, preventive measures, screen exposure time

## Abstract

Migraine remains a major source of disability in young adults, and the rapid rise of digital overuse introduces a modern environmental burden that may worsen cognitive impact. Emerging evidence suggests that prolonged screen engagement interacts with core migraine mechanisms, such as cortical hyperexcitability, trigeminovascular activation, and circadian disruption, through factors including high-intensity blue light, sustained near-work posture, and, more controversially, radiofrequency electromagnetic fields. This editorial calls for future research that should determine how digital environments heighten migraine vulnerability by integrating lifestyle factors with objective digital-use patterns, including photic load and sleep disruption. Early priorities should include testing blue-light reduction or ergonomic strategies and developing digital biomarkers, while long-term neuroimaging studies should clarify how sustained screen exposure alters cortical and thalamocortical dynamics. Clinically, these insights call for routine assessment of digital behaviors and the adoption of targeted digital-hygiene interventions such as blue-light filters, dark mode, screen-time limits, optimized posture, brightness adjustment, and scheduled sensory breaks as essential components of migraine care in addition to the pharmacological treatment.

## Editorial

Migraine is a pervasive condition of special interest among neurological disorders, by owning the second place in world’s cause of disability, through debilitating approximately 1 billion people worldwide [1]. It is characterized by recurrent attacks of moderate to severe headache, typically unilateral and pulsatile, accompanied by a constellation of sensory, autonomic, and cognitive symptoms, such as nausea, sensory hypersensitivity, cognitive slowing, and functional impairment. Physiopathologically, migraine represents not only a complex phenomenon that combines neural and vascular mechanisms, but also a brain network disorder involving cortical hyperexcitability, dysregulated sensory processing, altered pain modulation, and heightened vulnerability to environmental stimuli [2,3]. 

In recent years, the omnipresence of digital devices has introduced a new set of environmental exposures, which were correlated with various types of headaches, including migraine. As young adults represent the demographic with the highest daily screen time, they may face a disproportionate cognitive and clinical burden from digital overuse. For example, approximately 80% of young French adults own a smartphone and consume on average 3 hours per day of digital content. The negative impact of prolonged time spent watching digital content was demonstrated in various studies, among relevant results discovered being associated with higher BMI, psychiatric and sleep damaging (depression and insomnia), or substance abuse (cannabis). Emerging evidence suggests that these digital exposures may amplify key mechanisms implicated in migraine pathophysiology, including cortical and thalamic hyperexcitability, trigeminovascular activation, and circadian disruption [3,4]. According to the literature, there is a cluster of factors described that cause digital overuse-induced headache: high-intensity blue light, which is broadly studied, rapid visual refresh patterns, improper vertebral static leading to injury of cervical spine and muscles, heat, and visual stimulus from smartphones, and, finally, the emission of radiofrequency electromagnetic fields, which was inconclusive in recent studies (RF-EMFs) [1,4].

The mechanism through which blue-light exposure exacerbates or triggers migraine is demonstrated in several studies. Blue-light exposure overstimulates intrinsically photosensitive retinal ganglion cells, leading to increased activity in the trigeminovascular system and thus triggers migraine. Blue-light exposure alters sleep quality through suppression of melatonin production, causing a delay in the body's internal clock or circadian rhythm [1].

Although RF-EMFs are not supported by current evidence to be one of the primary causes of digital exposure-induced migraine, there are multiple mechanisms described regarding the headache induced by RF-EMF overexposure: destabilization of the brain’s electrical activity through disrupting transmission between neurons, or damage to the blood-brain barrier [4].

Prolonged smartphone usage can exacerbate migraine symptoms by increasing pain severity, altering sleep quality, and diminishing the response of medications. Some limits of previous studies conducted in this manner include the subjective nature of the scale used by self-reporting and the inability to quantify different associated factors and triggers of migraine, such as emotional stress, hormonal variability in women, light exposure, improper posture, and smoking patterns, necessitating further studies [1]. 

Future research must clarify the mechanisms through which digital environments amplify migraine vulnerability by approaching comprehensively various lifestyle and context factors, as well as subjective triggers associated with migraine, including but not limited to emotional stress, hormonal changes in women, poor posture, smoking habits, sleep schedule, and so on [1]. Likewise, in the near term, the development of digital biomarkers concluded from these types of studies, such as patterns of screen use, photic load, or sleep disruption captured via smartphones and wearables, could help in a more personalized migraine prediction and treatment plan. A second feasible line of inquiry is whether blue-light reduction strategies (e.g., via filters or green-light substitution), sensory-adaptive interface designs, or visual ergonomics produce measurable decreases in cortical hyperexcitability or attack frequency. In the long term, neuroimaging approaches such as functional magnetic resonance imaging (fMRI), electroencephalogram (EEG), and magnetoencephalogram (MEG) should be used to study how sustained visual and cognitive stimulation affects cortical excitability and thalamocortical network stability [2,3].

Treating migraine worsened by digital overuse requires abandoning vague lifestyle advice in favor of interventions grounded in further neurological understanding. Clinicians should incorporate a structured assessment of digital exposure - screen hours, brightness levels, evening use, multitasking behaviors, and ergonomics - into every clinical evaluation, just as systematically as sleep or stress history [1]. This evaluation can be addressed both subjectively through a semi-structured questionnaire about the environmental risk factors and triggers and objectively by measuring sleep quality, screen hour patterns, and brightness level with smartwatches and other devices [3]. Interventions should therefore include controlled sensory dosing: blue-light filters or dark mode (<450 nm emission), brightness reduction adjusted to ambient lighting, avoiding extreme contrasts, taking a 10-minute break every 50 minutes of visual work, or avoiding screen use for 2 hours before bedtime. Pharmacological treatments such as preventive therapies (e.g., topiramate, calcitonin gene-related peptide (CGRP) monoclonals) and acute treatments (triptans, gepants) remain essential, but they are insufficient unless paired with targeted digital hygiene practices, which should be further researched in clinical studies in order to be better defined as a medical term. This hybrid approach - pharmacological plus environmental modulation - should become the new standard of care for migraine in the digital era.

In conclusion, the widespread digitalization of daily life has introduced new challenges for managing migraines, particularly among young adults who are the heaviest users of screen-based technologies. The most immediate and achievable practice change is to systematically integrate digital-use assessment and guidance into routine migraine care. This means treating screen exposure - its duration, timing, posture, brightness, and cognitive load - as a modifiable clinical factor rather than a passive background behavior. Clinicians should begin asking targeted questions about daily digital habits, incorporate brief digital-hygiene counseling, and provide evidence-based recommendations such as limiting evening screen exposure, using blue-light filters, encouraging micro-breaks, and optimizing posture during device use. Migraine care in the digital era demands a new clinical mindset - one that recognizes digital exposure as a core environmental factor, not a peripheral inconvenience.
